# An Intervention With Michigan-Grown Wheat in Healthy Adult Humans to Determine Effect on Gut Microbiota: Protocol for a Crossover Trial

**DOI:** 10.2196/29046

**Published:** 2021-10-06

**Authors:** Gigi A Kinney, Eliot N Haddad, Linda S Garrow, Perry K W Ng, Sarah S Comstock

**Affiliations:** 1 Department of Food Science & Human Nutrition Michigan State University East Lansing, MI United States

**Keywords:** fiber, microbiota, whole grain, wheat, butyrate, calprotectin, lipocalin-2

## Abstract

**Background:**

Daily fiber intake can increase the diversity of the human gut microbiota as well as the abundance of beneficial microbes and their metabolites. Whole-grain wheat is high in fiber.

**Objective:**

This manuscript presents a study protocol designed to understand the effects of different types of wheat on gastrointestinal tract microbes.

**Methods:**

Human adults will consume crackers made from three types of wheat flour (refined soft white wheat, whole-grain soft white wheat, and whole-grain soft red wheat). In this study, participants will alternate between crackers made from refined soft white wheat flour to those made from whole-grain soft white wheat and whole-grain soft red wheat flour. Survey and stool sample collection will occur after 7-day treatment periods. We will assess how wheat consumption affects gastrointestinal bacteria by sequencing the V4 region of 16S rRNA gene amplicons and the inflammatory state of participants’ intestines using enzyme-linked immunosorbent assays. The butyrate production capacity of the gut microbiota will be determined by targeted quantitative real-time polymerase chain reaction.

**Results:**

We will report the treatment effects on alpha and beta diversity of the microbiota and taxa-specific differences. Microbiota results will be analyzed using the vegan package in R. Butyrate production capacity and biomarkers of intestinal inflammation will be analyzed using parametric statistical methods such as analysis of variance or linear regression. We expect whole wheat intake to increase butyrate production capacity, bacterial alpha diversity, and abundance of bacterial taxa responsive to phenolic compounds. Soft red wheat is also expected to decrease the concentration of inflammatory biomarkers in the stool of participants.

**Conclusions:**

This protocol describes the methods to be used in a study on the impact of wheat types on the human gastrointestinal microbiota and biomarkers of intestinal inflammation. The analysis of intestinal responses to the consumption of two types of whole wheat will expand our understanding of how specific foods affect health-associated outcomes.

**International Registered Report Identifier (IRRID):**

DERR1-10.2196/29046

## Introduction

### Background

An individual’s gut microbial community is colonized by trillions of microorganisms [1]. These microorganisms interact with other cells, influencing important metabolic and immune functions. In this way, the gut microbiota can benefit the host, offering protection from pathogens and supporting immune regulation [2]. However, a dysbiotic gut microbiota is linked to disturbances in metabolic and biological processes, leading to a variety of chronic diseases as well as cancers [3]. Individuals with reduced bacterial richness are characterized by phenotypes such as increased adiposity and insulin resistance [3].

Dietary intake is strongly associated with the composition of the gut microbiota [4-9]. This effect of diet on the gut bacterial community can occur within days, quickly switching among functional profiles characteristic of different diets, and thereby making food intake an important factor for managing the gut microbiota [5,10]. The Western diet is characterized by foods low in fiber and high in fat and refined sugars and carbohydrates [11]. The Western dietary pattern results in a gut microbiota characterized by decreased bacterial diversity with specific losses of important microbes such as *Bifidobacterium* spp., *Lactobacillus* spp., and Bacteroidetes [12-15].

As ultraprocessed foods have become a common component of the Western diet, resulting in a loss of whole grains and fiber, the gut microbial composition has become enriched with proinflammatory microbes that correlate with metabolic risk factors and which contribute to noncommunicable chronic diseases such as obesity and type 2 diabetes [14,16,17]. Consumption of whole grains, especially when used as a replacement for refined grains, improves the gut microbiota and is inversely associated with diseases characterized by a gastrointestinal microbial community in dysbiosis [18]. Whole grains have a prebiotic effect on the gut and restore diversity [12]. Prebiotics are compounds known to support the growth of beneficial intestinal microbes [19]. Bifidobacteria and lactobacilli respond positively to increased consumption of whole grains, as observed in a diet intervention study by Costabile et al [12]. Therefore, whole grain consumption may counteract the inhibitory effects of the modern Western diet on these bacterial genera [12,20] and, thus, may be one method to restore gastrointestinal microbiota diversity.

Grains are a staple of the Western diet; however, grains exist in many forms. Thus, it is important to consider the types of grains being consumed [21,22]. The 2015 and 2020 United States Department of Agriculture dietary guidelines for Americans recommended that half of all grains come from whole grains; nevertheless, refined grains are a trademark of the typical Western diet [14,23]. Compared with the consumption of whole grains, consumption of refined grains is associated with a higher risk of chronic disease. In rodent models where the animals are inoculated with human microbiota, low dietary intake of microbiota-accessible carbohydrates for several generations caused reduced microbial diversity [7].

Similarly, in humans, differences in the gut microbiota have been observed among different geographic groups consuming their culturally habitual diets [7,24]. Thus, over subsequent generations, refined grain intake can compound lost intestinal diversity and obliterate some taxa, most specifically Bacteroidales and Clostridiales [7]. Furthermore, the addition of microbiota-accessible carbohydrates to the murine diet suppressed a clinically important intestinal pathogen, *Clostridium difficile* [25]. Recent research has focused on replacing processed foods with whole foods to prevent this loss in microbial diversity and support gastrointestinal health [12,13].

Whole grains are rich in carbohydrates accessible to the gut microbiota, which bacteria can use for food and energy [26]. Consequently, adding whole grains to the diet leads to increased gut fermentation [15]. This process produces short-chain fatty acids, notably butyrate, an important anti-inflammatory and antioxidant molecule [15]. Diets rich in fiber have been positively associated with the presence of bifidobacteria and other lactic acid–producing bacteria and are associated with a shift toward long-term maintenance of metabolic and immune health [15]. Whole-grain wheat is a good source of fiber. Thus, increasing whole-grain wheat intake increases fiber intake, leading to a more diverse microbiome that not only contains the beneficial microbes themselves but also contains their metabolites [27].

Additional components of whole-grain wheat that convey health benefits are polyphenols [28]. Polyphenols act as antioxidants and prebiotics in the gut and, as such, interact with the gut microbial community affecting its composition [29]. When compared with the consumption of refined wheat, consumption of whole wheat increases the absorption of phytochemicals and the subsequent excretion of fecal ferulic acid, a polyphenol metabolite [13,30]. An increase in the availability of fecal ferulic acid through the feeding of whole grains has been associated with a significant increase in *Bacteroidetes* in the gut [13,20]. Thus, the positive relationship between whole grain ferulic acid and certain taxa of the gut microbiota is proposed as a possible mechanism for the alleviation of gut inflammation [13]. As different types of wheat have different polyphenolic content, it is important to understand how they differ in their relationship with the gut microbiota.

### Objectives

To fully understand the impact of whole-wheat products on humans, we must understand the effects of different types of wheat (ie, white vs red or hard vs soft) on human gastrointestinal tract microbes. However, previous studies have ignored these differences in wheat types. Therefore, we describe a protocol in which we will feed human adults crackers made from three different types of wheat flours and assess how cracker consumption affects (1) the gastrointestinal microbial communities and (2) the inflammatory state of the intestines. We hypothesize that whole-grain soft white wheat flour will promote different microbes compared with whole-grain soft red wheat flour. In addition, we expect that the whole-grain soft red wheat treatment will result in decreased intestinal inflammation compared with the refined white wheat flour treatment. The successful completion of the proposed research will improve our understanding of the unique effects of different types of wheat on the human gut microbiota and the inflammatory state of the gastrointestinal tract.

## Methods

### Design

**Figure 1:**
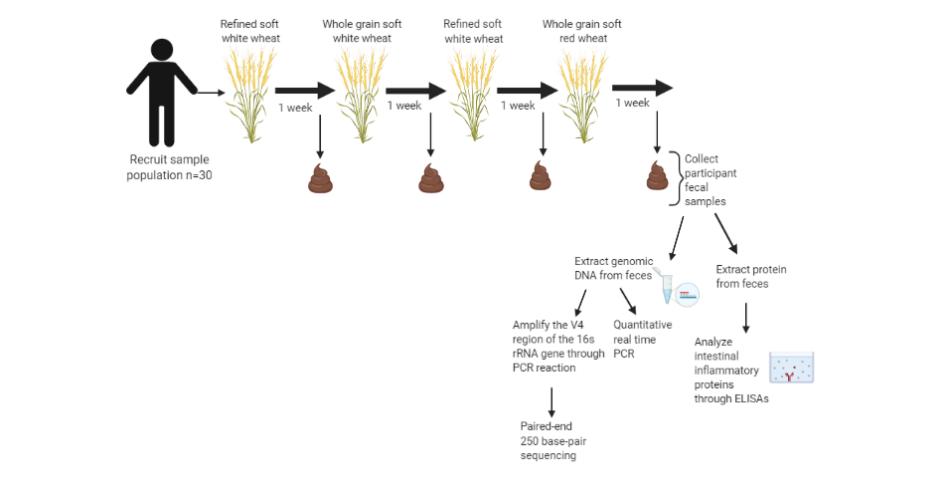
Overview of study design. ELISA: enzyme-linked immunosorbent assay; PCR: polymerase chain reaction.

This wheat intervention study will characterize the variable response of gut bacteria to supplementation in adult humans. Treatments will include (1) refined soft white wheat flour, (2) bran-containing whole-wheat flour of Michigan soft white wheat, or (3) bran-containing whole-wheat flour of Michigan soft red wheat. All 3 flour samples will be provided by King Milling Company (Lowell, Michigan). Each of the two whole-wheat interventions will be administered over a 1-week period, with a baseline week (refined soft white wheat flour) before the intervention begins and a washout week (refined soft white wheat flour) between the two test periods (Figure 1). Wheat flour will be administered to participants in the form of wheat crackers. The nutrient composition of the wheat crackers is provided (Table 1)**.** During each of the two test periods, 80 g of crackers made from whole-grain soft white wheat flour or whole-grain soft red wheat flour will provide each participant with approximately 10.7 g of total dietary fiber per day (Table 1). At the end of each 1-week treatment period, a stool sample as well as answers to a health and fiber intake questionnaire, will be collected from each participant. A total of 4 stool samples will be collected from each participant over the course of the study. Microbiome samples will be aliquoted and stored at −80°C until analysis.

**Table 1:** Nutrient composition of test crackers.

Nutrient composition			80-g portion of crackers		
			Refined soft white wheat flour crackers	Whole-grain soft white wheat flour crackers	Whole-grain soft red wheat flour crackers
Calories (kcal)			313	298	306
**Fat (g)**			6.44	7.16	8.00
	Saturated fat		1.68	1.82	2.08
	MUFA^a^		1.26	1.41	1.59
	PUFA^b^		3.16	3.54	3.92
	Trans fat		0.058	0.071	0.082
Protein (g)			7.15	9.10	9.35
**Carbohydrates (g)**					
	**Total dietary fiber**		3.99	10.72	10.72
		Insoluble fiber	1.54	7.54	7.35
		Soluble fiber	2.45	3.18	3.37
Sodium (mg)			439	452	454

^a^MUFA: monounsaturated fatty acids.

^b^PUFA: polyunsaturated fatty acids.

### Setting and Participant Recruitment

This study will be conducted during the summer in East Lansing, Michigan, on the campus of Michigan State University and will enroll 30 adults. Subjects will be recruited via flyers hung in buildings around the Michigan State University campus as well as through the Michigan State University–paid research pool [31]. To be included in the study, participants must be aged between 18 and 55 years as the gut microbiota matures by adulthood and remains stable throughout [1]. Participants must also have bowel movements at least once every 3 days, be willing to eat wheat crackers, and be available for weekly lab visits. Participants will be excluded if they take any nonsteroidal anti-inflammatory drugs, antacids, proton pump inhibitors, nutritional supplements, or multivitamins daily; have taken any antibiotics in the 2 weeks before the study; have gastrointestinal issues or diabetes; are pregnant; are following any special diet; or have food allergies. Participants will be provided with a US $50 cash incentive upon completion of the dietary intervention. All participants will provide written informed consent.

### Dietary Intervention

The dietary intervention will consist of a 4-week period of wheat consumption in the form of wheat crackers. Each week, participants will be given 7 bags of crackers (1 bag for each day of the week), containing approximately 80 g of baked crackers per bag, to be eaten throughout the following week. Each 80 g cracker allotment will provide approximately 10.7 g of total dietary fiber for crackers made from either whole-grain soft white flour or whole-grain soft red flour. The nutrients and calories of the 80 g cracker allotment consumed per day are listed in Table 1.

Crackers for each treatment week will be made in bulk to yield 260 daily portions (7 portions per participant plus extra), with approximately 80 g of baked crackers per daily portion per person. The cracker was formulated as 100 g flour (approximately 13% moisture), 1.5 g of iodized salt, 2 g of sugar, 6 g of vegetable shortening, and 49 to 50 g of tap water. The proportions of flour, salt, and sugar, according to the formulation stated above, will be added into the bowl of a Hobart mixer (model A-200) and whisked to combine the dry ingredients. Fat will be added to the dry ingredients and mixed until the fat particles are reduced in size to approximately 1 to 2 mm. Water will then be added and mixed until a firm dough forms. The amount of water (approximately 48 g) will need to be adjusted to ensure that all flour is fully hydrated. The dough will then be portioned by dividing it into 127 g aliquots based on the expected yield of 80 g of baked crackers per 127 g of dough (determined in preliminary testing). Each 127 g portion will be flattened between two pieces of parchment paper using a dough sheeter set to 3/32 inch. The top sheet of the parchment paper will be removed, and the dough will be cut into approximately 1- to 2-cm-wide strips. Crackers from each portion of dough will be baked in a carousel oven for approximately 11 to 12 minutes at 400 °F until browned. Crackers will be cooled on a rack on the workbench before being placed into individual resealable portion bags labeled *day 1* to *day 7*; then, 7 bags will be placed in one larger bag to be distributed to each participant.

**Figure 2:**
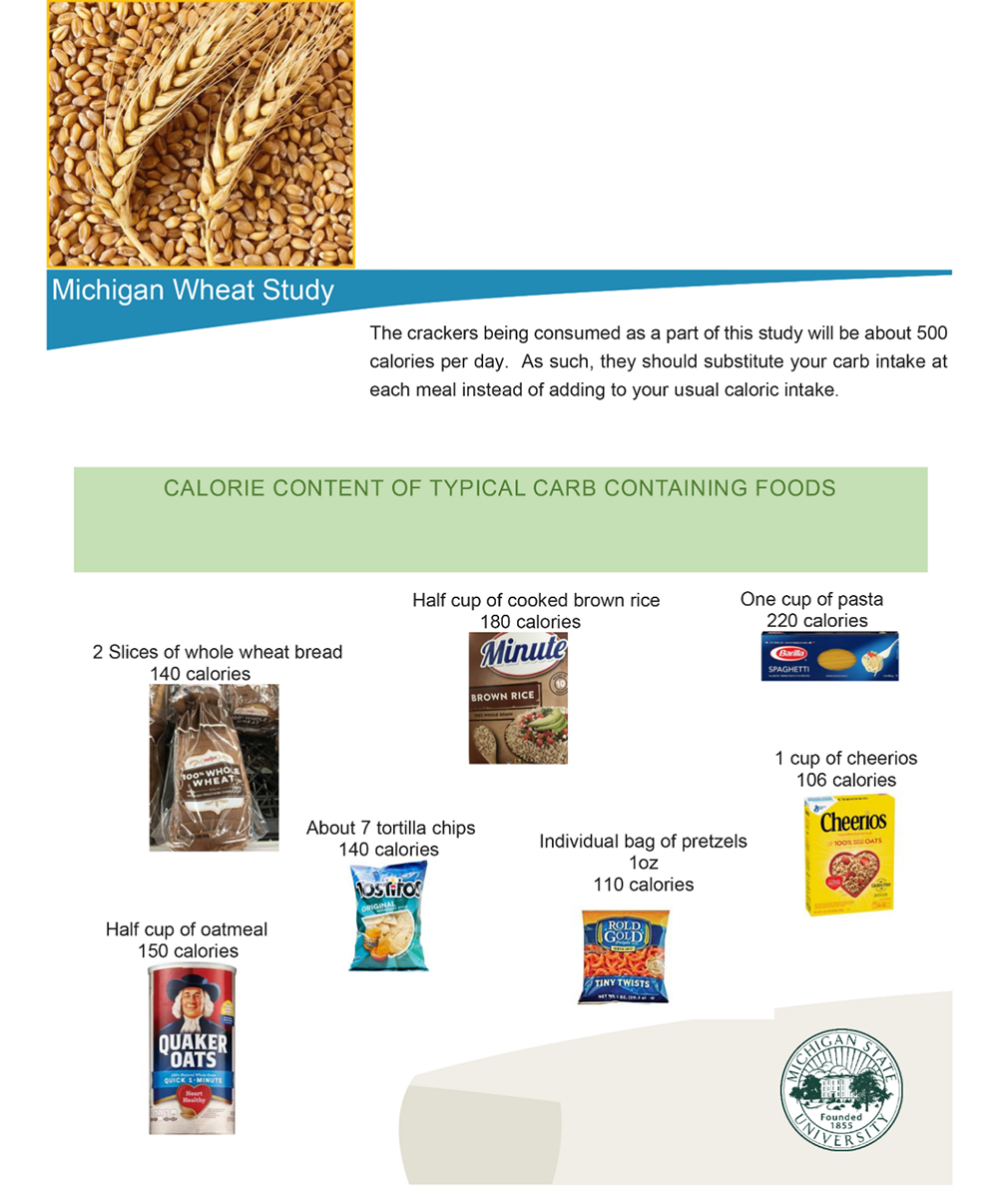
Flyer to be distributed to participants providing calories in common serving sizes of typical carbohydrate-containing foods.

As this study involves adding approximately 300 calories of carbohydrate-containing food to the daily diet, participants will receive flyers (Figure 2) informing them how to incorporate the crackers into their diets by replacing everyday dietary items such as cereals and bread with the test crackers. This will prevent or minimize participants’ weight gain.

### Outcomes

#### Overview

At the conclusion of each week, stool samples will be collected for analysis. The primary outcome measure will be the alpha diversity of the gut microbiota. This will be measured using the sequencing data obtained from the participants’ stool samples after DNA extraction and polymerase chain reaction (PCR) amplification of the V4 region of the 16S rRNA gene. The secondary outcome measure will be biomarkers of gastrointestinal inflammation, as measured by protein concentrations of calprotectin and lipocalin-2 in protein extracts of the stool samples. Furthermore, the microbiota beta diversity, abundance of *Bifidobacterium* and *Lactobacillus,* and butyrate production capacity of the gut microbiota will be assessed after each week of treatment. The abundance of *Bifidobacterium* and *Lactobacillus* will be determined from the sequencing results. The butyrate production capacity will be determined by quantitative real-time PCR assays targeting the bacterial butyrate kinase and butyrate transferase genes [32].

#### Other Measurements

Background characteristics of the study participants will be recorded during recruitment when each participant will complete a short questionnaire. In addition, current weight, health status, 24-hour dietary intake, and water source will be collected each week along with a fiber intake questionnaire (described below) recording the amount of fiber-containing foods consumed throughout the week. A 24-hour recall will be implemented as this has been previously reported to supply sufficient information to interpret microbiota data from participants’ fecal samples [33]. Participants will self-report their 24-hour dietary intake by free response to the question “What did you eat and drink in the past day? (Please give as much detail as possible, including the estimated amounts).”

### Data Analysis

#### Diet Analyses

##### Overview

Individual diet diversity scores will be calculated for each participant at each time point. The use of the diet diversity score provides a more general measure of the variety of dietary intake than specific food consumption data. This scoring system counts the number of food groups consumed by an individual. Data will be supplied based on the 24-hour intake data provided in response to this prompt, “What did you eat and drink in the past day? (Please give as much detail as possible, including the estimated amounts.)” as well as their answers to a checklist of 34 food and supplement items where participants are prompted to “Place an 'x' in the box next to anything that you ate in the past 24 hours.” The food checklist was designed to capture the intake of the foods listed in the diet diversity scoring tool document of the Food and Agriculture Organization of the United Nations [34]. Dietary diversity score will be assessed as described by the Food and Agriculture Organization of the United Nations [34].

##### Fiber Intake Analysis

The PhenX fiber intake protocol will be used to assess additional dietary fiber intake [35]. Each week, participants will receive a screener (PhenX protocol 50601) asking them to report how often they consumed different types of fiber-containing foods (ie, cereal, vegetables, and beans). Participants will record how often they consumed a portion of food—never, less than once a day, once a day, twice a day, three times a day, four times a day, or five or more times per day. A score will be assigned to each participant at each time point using the scoring methods provided by the PhenX protocol. This score will be used to account for the fiber in the diet that is not from the test crackers.

#### Microbiota Analyses

Stool samples will be extracted, prepared for sequencing of the 16S rRNA gene, and sequenced as described in references [36-38].

#### Butyrate Gene Analyses

Quantitative real-time PCR will be performed on the genomic DNA from each sample to determine the levels of butyryl-CoA: acetate CoA-transferase (*but*) and butyrate kinase (*buk*). Primer sets, But_RosEub, But_G.prausn, and Buk, previously designed by Vital et al [32], will be used. All genomic DNA samples will be diluted to 2.5 to 5 ng/μL. In a 96-well plate, genomic standards at concentrations of 10^2^ to 10^7^ will be used to create a standard curve. All wells will be filled with 7.5 μL SYBR Green, 3.5 μL ddH_2_O, 1 μL F primer, 1 μL R Primer, and 2 μL template. The thermocycling program to be performed is as follows: 2 minutes at 50°C, 10 minutes at 95°C, 45 seconds at 95°C, 45 seconds at annealing temperature for the specific primer, and 45 seconds at 72°C for 40 cycles. A one-way repeated-measures analysis of variance (ANOVA) will be performed on each set of data to compare the relative abundance and presence of the following butyrate synthesizers: *Faecalibacterium prausnitzii*, *Eubacteria *spp., *Roseburia *spp., and *Clostridium acetobutylicum*.

#### Intestinal Inflammatory Protein Analyses

Proteins will be extracted from the stool samples. The extraction buffers for lipocalin (catalog number 30757) and calprotectin (catalog number 30473) will be purchased from Epitope Diagnostics, Inc. First, the fecal samples will be aliquoted and stored at −80°C until protein extractions are performed. Then, approximately 100 mg of fecal material will be aliquoted into a conical tube, and the exact weight of the sample will be recorded. Each sample will be extracted separately with each of the two extraction buffers. Using a serological pipette, 4 mL of extraction buffer per 100 mg of stool will be aliquoted into a conical tube for each sample. Each tube will be gently vortexed and incubated at room temperature on an orbital shaker for 30 minutes. The protein extracts will then be aliquoted into microfuge tubes and stored at −80°C until ELISAs (enzyme-linked immunosorbent assays) are performed. The ELISA kits will be purchased from R&D Systems (a Bio-Techne brand) for human calprotectin (S100A8/S100A9; DS8900) and human lipocalin-2 (neutrophil gelatinase-associated lipocalin; DLCN20). Fecal extracts will be diluted at 1:25 or 1:100 in sample diluent for calprotectin assays and 1:20 or 1:100 in sample diluent for neutrophil gelatinase-associated lipocalin assays.

### Statistical Analyses

Statistical analysis of differences between pre- and posttests will be assessed using ANOVA or Friedman Wilcoxon rank-sum tests [39]. Multivariate statistics will be performed using R (R Foundation for Statistical Computing), a free statistical software program. To compare the alpha diversity of the microbial communities, Chao1 (a measure of richness), inverse Simpson (a measure of richness and evenness, with emphasis on evenness), and Shannon diversity (a measure of richness and evenness, with emphasis on richness) will be calculated in R using the vegan package [40]. ANOVA and Wilcoxon signed-rank test or Friedman test with post hoc comparisons will be used to compare alpha diversity across treatment categories after intervention [41]. For beta diversity, Bray-Curtis and Sorenson distances will be used with principal coordinates analysis ordination to produce plots of the distances between samples. Differences in beta diversity between time points will be determined using permutational multivariate ANOVA (PERMANOVA) via the adonis function in the vegan package, and differences in group dispersion will be determined using PERMDISP [40]. PERMANOVA may also be used to determine the relationship between the distance metrics and wheat variety, as well as other variables. Metastats (a function of the mothur software package) will be performed on the subsampled abundance data to test for significant differences in operational taxonomic unit abundance between groups [42]. Changes in taxa (both genus and phylum level) >1% average relative abundance will be determined across time points using the Friedman test with post hoc comparisons. Where appropriate, the negative binomial and zero-inflated mixed models in the R package will be used to test for differences in taxa abundance over time [43].

Enterotypes will be manually assigned based on clustering within quadrants in a Bray-Curtis principal coordinates analysis plot overlaid with the most abundant taxa driving community differences, as in Wu et al [4], using the envfit function in the vegan package. Significant differences between enterotypes will be determined using ANOVA for parametric data and the Kruskal-Wallis test for nonparametric data. Differences between time points within each enterotype will be determined using the Friedman test with post hoc comparisons.

### Response Analysis

If only a subset of participants is found to respond to the treatment, response groups will be determined by an individual’s change in alpha diversity metrics across the treatments. A participant will be considered a *positive responder* if alpha diversity (either Chao1, Shannon, or inverse Simpson) is greater at time points B and D (whole wheat) than at both A and C (refined white wheat). However, if this change is not observed, participants will be considered *nonresponders*. After post hoc assignment of response groups, differences in overall alpha diversity between positive responder and nonresponder groups will be tested with ANOVA (if parametric) or Wilcoxon ranked-sum (if nonparametric). The normality of data will be determined using the Shapiro-Wilk test. Differences will be tested within each time point. Beta diversity differences between response groups will be determined at each time point using PERMANOVA and PERMDISP. Continuous demographic variables (age, BMI, and fiber intake) will be compared with a two-tailed *t* test if parametric and a Wilcoxon rank-sum test if nonparametric, with normality confirmed using the Shapiro-Wilk test. The Fisher exact test will be used to determine the independence of sex, smoking exposure, and race regarding response to treatment. Differences in genera present at >1% average relative abundance will be tested between response groups using a negative binomial model in the MASS package [44].

## Results

The study procedures were approved by the Michigan State University institutional review board (IRB #00002638) in spring 2019. Sample collection occurred in summer 2019. The results of this research are expected to be published in late 2021.

## Discussion

### Impact

The primary aim of this study is to measure the alpha diversity of gut microbiota following each week of cracker treatment to determine whether whole-wheat consumption increases alpha diversity. Additional aims are to measure the levels of gastrointestinal inflammation as well as *Bifidobacterium*, *Lactobacillus,* and butyrate-producing bacteria that are present following each treatment. These analyses will elucidate how different types of wheat may affect the gastrointestinal tract microbiota.

Only recently have we begun to understand the mechanism by which whole grains promote health [12,16,21,45]. Various microbes and metabolites have been hypothesized to decrease inflammation; however, much is still unknown [14]. Our aim—to measure how microbial diversity and levels of certain bacteria are affected by whole-wheat intake—will elucidate the potential mechanisms by which whole grains affect health. In addition, little is known about the effects of different types of wheat (eg, white vs red) on the gut microbiota. Specific wheat types have different molecular compositions and may therefore promote different microbes [46-49]. Taking into account the effects of different types of whole wheat will allow us to understand better the food characteristics that underlie the impact of whole-wheat consumption on humans.

We anticipate that the results of this intervention study will have implications for both wheat producers and consumers. Recent trends suggest an increase in the number of consumers who avoid wheat products as part of new diets that emphasize carbohydrate reduction, despite the unclear benefits of avoidance in those without celiac disease [50]. In fact, the risks of avoiding gluten, and subsequently whole grains, include deficiency of micronutrients, fiber, and an increased consumption of refined products, which may outweigh the perceived benefits of avoiding wheat [50]. Understanding how microbes and gastrointestinal inflammation are affected by the consumption of whole-grain wheat may increase the consumption of whole-grain wheat by health-conscious consumers.

### Strengths and Weaknesses

An important strength of this study is the composition of the crackers. The aim of the study is to test the effect of specific types of wheat; thus, it is important to note that we have created a cracker formulation that is composed almost entirely of the wheat of interest with few additional ingredients. The simple recipe we have developed for the crackers will improve confidence that the differences observed in the microbial community and inflammatory biomarkers are owing to the consumption of the wheat in the study crackers rather than a different ingredient in the treatment food. The quick turnaround from the cracker intake to sample collection also helps strengthen the study. Stool samples will be collected on the final day of each week's cracker intake, ensuring that little time has elapsed between the last cracker intake and stool sample collection. This is important for microbiota analyses as the temporal intake of food is tightly associated with microbial composition [51]. In addition, we will allow sufficient time to pass between each intervention through the use of a washout period. Johnson et al [33] described a minimum 3- to 5-day period between crossing-over diets to allow the microbiome to return to baseline. The crossover design of this study will allow us to perform within-person comparisons, placing less emphasis on interindividual differences in the gut microbiome [33].

A potential limitation of our study may be participant retention. Participants will be expected to consume approximately 80 g of crackers every day for 4 weeks, so it is important that they enjoy the taste of the crackers to ensure their compliance and continuance in the study. However, the study participants may tire of the taste of the crackers. A high dropout rate could hinder our ability to detect statistically significant differences.

Whole grains alleviate dysbiosis in the gut microbiota; however, little is known about the impact of different wheat types on this phenomenon [12,18,20]. A 4-week wheat intervention to investigate the effect of different types of Michigan-grown wheat on the gut microbiota is described in this protocol. The results of this study will improve our understanding of the unique effects of different types of wheat on gut microbiota and on the inflammatory state of the human gastrointestinal tract.

## Abbreviations

ANOVA: analysis of variance
ELISA: enzyme-linked immunosorbent assay
PCR: polymerase chain reaction
PERMANOVA: permutational multivariate analysis of variance
